# Biosynthesis and Its Regulatory Mechanisms of 2-(2-Phenylethyl)chromones in Agarwood

**DOI:** 10.3390/plants14071012

**Published:** 2025-03-24

**Authors:** Wenli Wu, Xiaoyang Jiang, Luyuan Jiang, Iain Wilson, Fenjuan Shao, Deyou Qiu

**Affiliations:** 1 State Key Laboratory of Tree Genetics and Breeding, Key Laboratory of Tree Breeding and Cultivation of National Forestry and Grassland Administration, Research Institute of Forestry, Chinese Academy of Forestry, Beijing 100091, China; wwlarroyo@163.com (W.W.); xiaoyang9612@126.com (X.J.); qiudy@caf.ac.cn (D.Q.); 2 CSIRO Agriculture and Food, Canberra, ACT 2601, Australia; iain.wilson@csiro.au

**Keywords:** 2-(2-phenylethyl)chromones, structure and function, biosynthesis pathway, regulatory mechanisms

## Abstract

Agarwood, a highly prized traditional medicinal material and natural spice, holds significant economic and medicinal value. Widely utilized as a fragrant agent, it is also employed in the treatment of diverse ailments, including rheumatism, fever, asthma, bronchitis, cancer, and gastrointestinal or reproductive disorders. These functions are primarily attributed to the accumulation of 2-(2-phenylethyl)chromones (PECs), a class of bioactive compounds. In recent years, PECs have emerged as critical components in the development of agarwood-derived pharmaceuticals and commercial products, garnering substantial scientific attention. This review consolidates current advancements in the structure and function of PECs and examines and discusses the structural genes and regulatory transcription factors associated with PECs biosynthesis. By synthesizing this knowledge, this review establishes a foundation for elucidating the complete biosynthetic pathways and regulatory mechanisms governing PECs production, thereby facilitating future research and applications.

## 1. Introduction

Agarwood is a resin-embedded wood produced in response to injury or infection in *Thymelaeaceae* plants and is predominantly found in the southern provinces of China and Southeast Asian countries, including Indonesia, Malaysia, Vietnam, Thailand, and India [1,2,3]. Revered since ancient times as the “gold in medicine” and the “king of fragrance”, agarwood has a rich history documented as early as the Xijing Magazine of the Han Dynasty. Subsequent records in texts like *Nanzhou Exotica Zhi*, *Famous Physician’s Record*, *Ben Cao Tu Jing*, *Incense Multiplier*, and *Ben Cao Gang Mu* highlight its aromatic and medicinal significance. Agarwood is highly valued for its aromatic properties and medicinal value, including the treatment of stomach cold, vomiting, angina, neurasthenia, nausea, and general soreness [4]. Beyond medicine, agarwood has a prominent role in health care, perfumery, and other industries [5].

The distinctive aroma of agarwood arises from the secondary metabolites found primarily in the stems of *Thymelaeaceae* species. Among these metabolites, terpenoids and 2-(2-phenylethyl)chromones (PECs) stand out as the most bioactive compounds. Due to their medicinal and aromatic importance, PECs have garnered significant attention. As characteristic constituents of agarwood, PECs contribute significantly to its fragrance and pharmacological properties [6]. Studies reveal significant differences in PEC content across species, with Chi-Nan agarwood showing levels of 2-(2-phenylethyl)chromone and 2-[2-(4′-methoxyphenyl)ethyl]chromone that are 170 and 420 times higher, respectively, than in ordinary agarwood [7]. These variations are believed to influence the quality and variety of agarwood, with PECs identified as a key factor [8,9,10]. Additionally, PECs exhibit diverse biological activities, including cytotoxicity, neuroprotection, acetylcholinesterase inhibition, and antioxidant, anti-inflammatory, and bacteriostatic properties [10,11,12,13,14,15].

Given the importance of PECs as a quality indicator, enhancing their content in cultivated agarwood species is a significant goal for breeding and synthetic biology. A comprehensive understanding of PECs’ structural diversity, biosynthetic pathways, and regulatory mechanisms is critical to achieving this objective. This review aims to summarize recent advances in PEC research, providing a foundation for future studies that will elucidate the complete biosynthetic pathways and regulatory mechanisms of PECs, thereby paving the way for improved agarwood production.

## 2. Structure and Function of PEC

**Figure 1:**
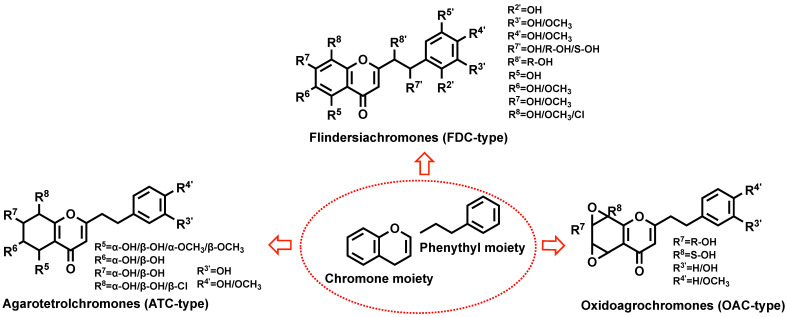
The core structure of chromone and 3 kinds of 2-(2-phenylethyl)chromone structural formula.

PEC is a C-2 phenethyl substitute for chromone, with the parent nuclear backbone of chromone (Figure 1) and similar substituents (hydroxyl and methoxy dominant). The changes of substituents lead to chromone kinds and structural diversity. According to the different backbone chains of PECs, PECs can be mainly classified into three categories, including flindersiachromones (FDC-type), oxidoagarochromones (OAC-type), and agarotetrolchromones (ATC-type) (Figure 1).

### 2.1. FDC-Type

FDC-type PECs constitute a structurally diverse and pharmacologically significant subclass of agarwood chromones. Their archetypal representatives, such as PEC and 2-[2-(4-methoxyphenyl)ethyl]chromone [16,17], exemplify the characteristic substitution patterns that underpin their bioactivity. To date, over 80 FDC-type derivatives have been structurally characterized [16,17], revealing a conserved preference for hydroxyl or methoxy substitutions at C-6, C-7, C-5, and C-8 positions. Notably, methoxy groups exhibit a pronounced propensity for C-7 (hydroxyl counterparts of substitution frequencies), while chlorine substituents occasionally occupy C-7/C-8, suggesting evolutionary pressure for halogenation in specific ecological niches.

The pharmacological profile of FDC-type PECs demonstrates remarkable polypharmacology: neuroprotection via Nrf2/ARE pathway modulation, competitive acetylcholinesterase (AChE) inhibition [18], and tyrosinase suppression [18]. Their antitumor efficacy, particularly against HepG2 and A549 cell lines [18], correlates with PPARγ partial agonism [19], implicating metabolic regulation in their cytotoxic mechanisms. The structural basis for their anti-inflammatory activity likely resides in the ortho-dihydroxy motif’s radical scavenging capacity.

### 2.2. ATC-Type

The ATC-type PECs, exemplified by agarol, represent a distinct class with over 40 reported derivatives [17]. Their structural hallmark lies in stereochemically complex oxidation patterns at C-5–C-8, predominantly featuring axial-equatorial hydroxyl/methoxy configurations that dictate three-dimensional pharmacophore topology. Molecular dynamics simulations suggest these stereochemical variations significantly impact target engagement, particularly in AChE inhibition.

While sharing partial overlap with FDC-type bioactivities (e.g., AChE inhibition and anti-inflammatory action), ATC derivatives exhibit unique structure–activity relationships. Their antitumor cytotoxicity [18] appears contingent on C-7 methoxylation and C-8 hydroxylation, showing the nuanced role of oxygenation patterns in differentiating target specificity between PEC subclasses.

### 2.3. OAC-Type

OAC-type PECs, characterized by A-ring oxiranyl groups, remain the least explored subclass, with only three characterized derivatives [17,18]. Their oxidation patterns at C-5–C-8 combine epoxide bridges [17] with stereoselective hydroxyl/methoxy substitutions, exclusively at C-5/C-8 for methoxy groups. The absence of phenethyl substituents, replaced by C-4′ methoxy or C-3′ hydroxyl groups, creates distinct electronic profiles that may enhance blood–brain barrier permeability relative to other PECs.

Despite structural novelty, OAC bioactivities currently mirror ATC-type profiles, including AChE inhibition [18] and antibacterial action [18]. However, preliminary molecular docking suggests their epoxy groups may enable covalent target modification—a mechanism absent in other subclasses. This understudied reactivity warrants investigation through proteomic profiling to elucidate potential unique therapeutic applications.

## 3. Biosynthetic Pathways of PEC

The biosynthesis of PECs represents a complex interplay of enzymatic cascades and regulatory networks; yet significant lacunae persist in our understanding of its full mechanistic trajectory. While pivotal enzymes such as PEC precursor synthase (PECPS) and *A. sinensis* polyketide synthase (AsPKS)—a type III PKS that catalyzes the iterative decarboxylative condensation of malonyl-CoA units to construct polyketide scaffolds—have been implicated in early pathway modulation [20,21,22], debates persist regarding the functional plasticity of PKS enzymes. Some studies emphasize their broad substrate promiscuity, enabling divergent metabolic outcomes, while others propose stricter substrate-channeling mechanisms. The complete biosynthetic sequence remains fragmented. The foundational architecture of PECs originates from phenylalanine-derived intermediates, which undergo iterative elongation via PKS-mediated condensation—a hallmark reaction where these multidomain enzymes orchestrate carbon–carbon bond formation through acyltransferase and ketosynthase activities, followed by regioselective hydroxylation and O-methylation modifications [10,16,20,23]. Notably, Okudera and Ito [24] proposed a bifurcated pathway wherein di-epoxy-tetrahydro-2-(2-phenethyl)chromone serves as the central precursor, undergoing stereochemical rearrangements to yield flidersia-type derivatives, a study later supported by Dai et al. [6,25]. This underscores the metabolic promiscuity of chromone scaffolds, which emerge as convergence points for divergent pathways, including the pentaketone pathway and the shikimic acid pathway [26]. Contemporary evidence positions type III PKS, *O*-methyltransferases (OMTs), and glycosyltransferases as enzymatic linchpins, though their substrate specificities and spatiotemporal regulation remain inadequately characterized.

The PKS family, particularly type III isoforms, has emerged as a critical determinant of PEC structural diversity. Divergent perspectives exist on whether this diversity stems from inherent PKS multifunctionality or tight regulatory control over discrete isoforms. Liao et al. [16] elucidated a dual catalytic role for PKS in orchestrating the condensation of dihydrocinnamoyl-CoA and malonyl-CoA with aromatic acyl-CoA derivatives, thereby generating the PEC skeleton essential for flidersia-type PEC cyclization. However, Wang et al. [22] observed strict transcriptional specificity of AsPKS1/2 under abiotic stress, suggesting context-dependent functional specialization. Intriguingly, these authors demonstrated that 6-hydroxyl group incorporation—a hallmark of bioactive PECs—requires precise co-ordination between type III PKS and cytochrome P450 hydroxylases, suggesting a co-evolutionary mechanism for scaffold diversification. Subsequent work by Wang et al. expanded this paradigm, revealing that abiotic stressors (e.g., salinity and cadmium) and phytohormones (e.g., methyl jasmonate) differentially modulate *AsCHS1*, *AsPKS1*, and *AsPKS2* transcription, thereby implicating PKS in both metabolic and stress-adaptation roles [22]. These findings align with Chen et al. [27] correlation between AsCHS expression and PEC accumulation in agarwood, though conflicting interpretations exist; the absence of in planta enzyme kinetic data leaves open questions about whether PKS activity represents a rate-limiting bottleneck or a permissive “gate-opener” in pathway flux. A seminal breakthrough by Wang et al. [20] identified PECPS as the gatekeeper enzyme for diarylpentanoid biosynthesis, with heterologous AsPECPS expression in *Nicotiana benthamiana* conferring abiotic stress tolerance (salt and ABA stress [28])—a finding that bridges secondary metabolism with ecological fitness [28]. Notably, Xiao et al. challenged strict pathway orthodoxy by demonstrating that AsPKS3~5 could catalyze the synthesis of 1-methyl-2-phenethylquinolin-4(1H)-one—a structural analog of PECs—implying broader catalytic versatility than previously ascribed to type III PKSs [21] (Figure 1). Further complexity arises from Ding et al. [29] identification of tandem-duplicated AsPKS07/08 paralogs lacking orthologs in model systems, which transiently reconstitute PEC biosynthesis in *N. benthamiana*—a system ripe for combinatorial enzymology studies to resolve whether these paralogs exhibit neo-functionalization or substrate redundancy.

Structural diversification of PECs is critically dependent on post-PKS tailoring enzymes, particularly OMTs and cytochrome P450s (CYPs), though their interplay remains enigmatic. While Wang et al. [10] nominated three OMTs (*flavonol-OMT1*, *flavonol-3-OMT*, and *caffeoyl-CoA-OMT*) as putative PEC methylators, functional validation has not been carried out. Yang et al. [30] mechanistically linked ethylene-induced *AsOMT1* upregulation to oxymethyl-chromone production, while Wu et al. [31] resolved the regiospecificity of AsOMT11/16 in 6-methoxy-2-(2-phenylethyl)chromone formation. Contrarily, Wang et al. [32] revealed AsOMT1’s dual substrate flexibility, catalyzing both caffeoyl-CoA methylation and non-regioselective chromone modifications—a plasticity that may underpin chemodiversity in stress responses. For CYPs, despite Das et al. [23] genome-wide identification of *Aquilaria* P450s potentially associated with PEC hydroxylation, the absence of in vitro reconstitution or gene knockout evidence leaves their roles speculative. A nascent frontier involves glycosyltransferases, with UGT71BD1 emerging as a multi-substrate catalyst capable of decorating PECs with diverse glycones [33]. This discovery not only expands the toolkit for synthetic biology but also raises evolutionary questions about glycosyltransferases recruitment into specialized metabolism.

Collectively, these advances illuminate a dynamic, multi-layered biosynthetic framework for PECs; yet persistent knowledge gaps—particularly regarding CYP/GT functional characterization, pathway crosstalk, and transcriptional regulation—underscore the need for systems-level omics integration and CRISPR-based functional genomics.

## 4. Regulation of PEC Biosynthesis

**Figure 2:**
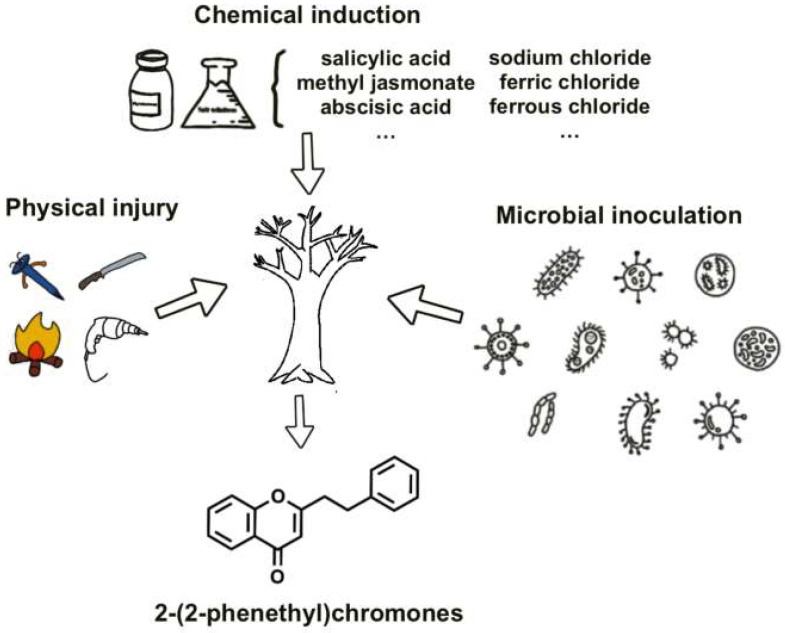
Induction methods for forming agarwood.

The formation of agarwood in *A. sinensis* represents a sophisticated defense response triggered by both endogenous molecular regulation and exogenous environmental stressors. This complex interplay co-ordinates the timed accumulation of pharmaceutically valuable PECs and sesquiterpenes through multiple regulatory layers. While artificial induction techniques—including physical wounding, chemical elicitation, and microbial inoculation—have been developed to accelerate resin deposition, their industrial implementation faces critical limitations [28,34,35] (Figure 2). These methods frequently yield inconsistent PEC profiles, induce xylem degradation, and produce chemically inferior agarwood compared to natural agarwood [36,37]. Such technical constraints stem fundamentally from our incomplete understanding of the molecular regulation governing PECs biosynthesis, particularly the transcriptional networks coordinating this specialized metabolism.

Emerging evidence positions transcription factors (TFs) as central regulatory nodes in PEC biosynthesis, operating beyond the well-characterized enzymatic machinery (PKSs/OMTs). Comparative analyses with model plants reveal conserved TF families—MYB, bZIP, and WRKY—as evolutionary architects of plant secondary metabolism, typically through cis-element binding and promoter activation [38]. In *Aquilaria* genus, preliminary studies identify putative bZIP regulators (AsbZIP14/41), potentially activating AsPKS genes via ACGT-motif interactions [29,39,40]. Contrastingly, MYB TFs like AsMYB054 demonstrate repressive effects on AsPKS02/09, suggesting a regulatory counterbalance mechanism to prevent metabolic overflow—a phenomenon observed in Isoprenoid pathways [41,42]. The expression analysis of 15 *AsNACs* in *A. sinensis* under injury treatment revealed that *AsNAC019* and *AsNAC098* exhibited positive correlations with four *PKS* genes and functionally activated the transcription of *AsPKS07* through promoter binding [43]. This duality of activation–suppression networks imply sophisticated metabolic gatekeeping, yet critical knowledge gaps persist.

Three fundamental challenges hinder progress. First, the putative TF–gene regulatory relationships remain unvalidated due to insufficient functional studies (e.g., CRISPR knockouts/TF overexpression). Second, the spatial organization of PEC biosynthetic enzymes—whether forming transient metabolons or operating through diffusive mechanisms—directly impacts pathway efficiency but remains unresolved. Third, while artificial induction methods (chemical/nailing/fungal inoculation) empirically activate PEC synthesis [44,45,46], the molecular cascades linking these stressors to TF network activation are poorly mapped. This disconnects between empirical practice and mechanistic understanding explains why current methods achieve only partial success—chemical inducers like methyl jasmonate effectively boost PECs yields (>60% content) [24,45] but inadvertently trigger xylem decay [36].

Addressing these limitations requires paradigm-shifting approaches. Single-cell metabolomics could resolve the spatiotemporal co-ordination of PEC synthesis, while CRISPR-Cas9-mediated TF manipulation may establish causal regulatory relationships. Furthermore, comparative transcriptomics of chemically induced vs. naturally stressed trees might identify conserved TF activation patterns, enabling smarter induction protocols that mimic ecological stress signatures without collateral damage. Given that PEC composition determines commercial agarwood quality [21], decoding these regulatory hierarchies is not merely academically significant but essential for developing sustainable, high-yield production systems. The integration of functional genomics with improved induction technologies promises to bridge the artificial–natural quality gap while mitigating current environmental concerns like heavy metal accumulation from chloride-based elicitors [36,44].

This synthesis reveals that advancing agarwood biotechnology necessitates shifting from phenomenological induction methods to mechanism-driven regulatory engineering—a transition requiring concerted exploration of both transcriptional networks and their environmental activation triggers.

**Figure 3:**
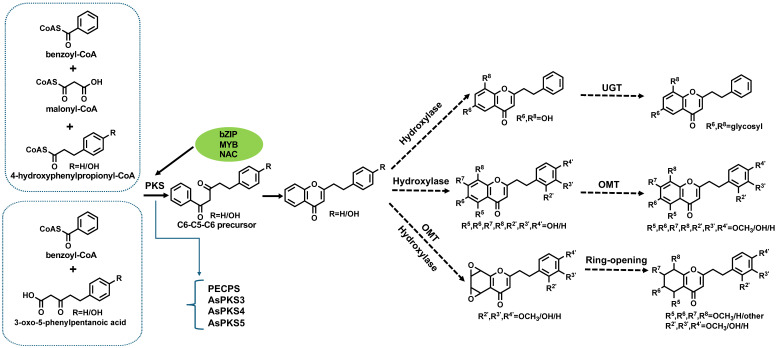
PEC biosynthesis pathway.

Based on the results from the literature, we believe that the biosynthesis of PECs mainly involves three key steps (Figure 3): (1) the enzymes involved in the reaction of 2-(2-phenethyl)chromone backbone are type III polyketide synthase and cyclases [17]; (2) the process of generating other types of chromone from 2-(2-phenethyl) chromone, which involves enzymes such as cyclooxygenase, hydroxylase, and reductase, among which the enzymes of the hydroxylase (CYPs) are the most abundant; (3) the enzyme involved in the process of generating methylated PECs may be OMTs. The formation of 2-(2-phenethyl)chromone backbone is the first and the most important step. At present, the function of type III polyketide synthase and several OMTs has been verified, which provides an important basis for further elucidation of the biosynthesis pathway of PECs. Moreover, polyketide synthase, OMTs, and hydroxylase also play a crucial role in the biosynthesis of PECs. However, more direct confirmatory evidence is lacking. In addition, studies on regulatory factors associated with the biosynthesis pathway of PECs are also lacking.

## 5. Application of Multi-Omics in Agarwood

The convergence of high-throughput omics technologies has revolutionized phytochemical investigations in *Aquilaria* species, particularly in deciphering the complex biosynthetic pathways of PECs. Pioneering work by Li et al. [2] established critical mass spectrometry frameworks through UPLC–ESI-QTOF-MS analysis, revealing 14 diagnostic metabolic markers, including 6,7-dimethoxy-2-(2-phenethyl)chromone and its structural analogs. This seminal study not only differentiated wild versus cultivated agarwood but also underscored the chemical plasticity of PEC biosynthesis under environmental pressures. Nguyen et al. (2017) employed a combination of 1H-NMR spectroscopy, gas chromatography–mass spectrometry (GC-MS), and DNA-based molecular marker analysis to authenticate the botanical origins of *Aquilaria crassna* and *Aquilaria malaccensis* [47].

Recent genomic breakthroughs have provided unprecedented resolution for understanding chromone metabolism. The chromosomal assemblies by Ding et al. [48] (726.5 Mb genome, 29,203 genes) and Nong et al. [49] (783.8 Mb genome, 35,965 genes) reveal striking genomic heteromorphism, particularly in repetitive element distribution (59.13% vs. 48.2%, respectively). These structural variations likely influence PEC biosynthesis gene clusters. The integration of long-read nanopore sequencing with Hi-C scaffolding may be particularly valuable for resolving complex biosynthetic pathways of PECs.

Transcriptomics further illuminate stress-responsive biosynthesis regulation. Xu et al. [50] identified 89,137 unigenes through 454 pyrosequencing, revealing wound-induced expression cascades in *A. sinensis*. Subsequent work by Ye et al. [51] delineated spatial expression gradients across agarwood formation zones, identifying 83,467 unigenes. Tu’s group [10,52] extended these findings through salt-stress experiments, demonstrating ROS-mediated regulation via NADPH oxidases (*AsRbohA-C*)—a mechanism evolutionarily conserved in plant specialized metabolism. Their identification of 18,069 differentially expressed transcripts underscores the pleiotropic effects of environmental stressors on secondary metabolism.

Proteomic insights by Gao et al. [53] complement these nucleic-acid-level findings, with iTRAQ analysis revealing 504 differentially expressed proteins including sesquiterpene synthases and stress-response mediators. The co-ordinated upregulation of superoxide dismutase with sesquiterpene production suggests redox homeostasis plays a gatekeeping role in resin genesis—a hypothesis supported by recent systems biology models.

While current multi-omics approaches have substantially advanced agarwood research, significant knowledge gaps persist. The discordant genome assemblies (726.5 vs. 783.8 Mb) highlight technical challenges in resolving complex plant genomes. Furthermore, the limited integration of metabolomic networks with genomic data hinders pathway elucidation—a challenge addressable through machine learning approaches. Future research must bridge these technological and conceptual divides to fully exploit multi-omics synergies. The comprehensive application of metabolomics, genomics, and transcriptomics provides a robust foundation for future research on functional gene cloning and the biosynthetic pathways of PECs in agarwood.

## 6. Conclusions and Perspective

PECs, as signature phytochemical constituents of agarwood, exhibit multifaceted biological activities encompassing cytotoxicity, neuroprotection, acetylcholinesterase inhibition, and antioxidant, anti-inflammatory, and bacteriostatic effects. This review systematically consolidates advancements in the structure and function, biosynthetic pathways, and regulatory mechanisms of PECs. Despite extensive preliminary investigations, critical gaps persist in the current understanding of PECs. Notably, the absence of comprehensive knowledge of biosynthetic pathways hinders their translational potential. Furthermore, while induction techniques such as physical wounding and chemical elicitation have been employed to stimulate agarwood formation, the molecular interplay between these induction methods and PECs biosynthesis remains inadequately characterized.

Addressing these challenges necessitates a paradigm shift toward interdisciplinary integration. First, leveraging multi-omics platforms—including genomics, transcriptomics, proteomics, and metabolomics—will be pivotal for mapping the biosynthetic network of PECs and identifying rate-limiting enzymes. Advanced molecular bioinformatics tools could facilitate the discovery of transcription factors and epigenetic regulators governing PECs synthesis under stress conditions. Second, synthetic biology offers transformative potential for scalable PECs production. Heterologous expression of biosynthetic gene clusters in microbial chassis, coupled with CRISPR-based metabolic engineering, could circumvent the ecological and technical limitations of traditional agarwood harvesting. Concurrently, elucidating the ecological and developmental triggers of agarwood formation—such as the role of microbial symbionts or stress-responsive phytohormones—will inform sustainable cultivation strategies. Such advancements could revolutionize agarwood quality control, enable precision breeding of high-yield cultivars, and align with global conservation efforts. Ultimately, bridging these scientific and technological gaps will not only deepen the understanding of PECs as bioactive entities but also position agarwood as a sustainable resource for pharmaceutical, cosmetic, and agricultural industries. Collaborative efforts across academia and industry are essential to translate these insights into economically viable and ecologically responsible innovations.

## Abbreviations

PECs	2-(2-Phenylethyl)chromones
FDC-type	Flindersiachromones
OAC-type	Oxidoagarochromones
ATC-type	Agarotetrolchromones
AChE	Acetylcholinesterase
PDE	Phosphodiesterase
PKS	Polyketide synthase
OMTs	O-methylytransferases
PECPS	PECs precursor synthase
CYPs	Cytochrome P450 enzymes
TFs	Transcription factors
Bzip	Basic leucine zips
UPLC–ESI-QTOF-MS	Electrospray ionization mass spectrometry

## References

[1] Lei Z.D., Liu D.L., Zhao Y.M., Gao X.X. (2018). A new 2-(2-phenylethyl)chromone from *Aquilaria sinensis*. Chem. Nat. Compd..

[2] Li Y.B., Sheng N., Wang L.L., Li S.J., Chen J.N., Lai X.P. (2016). Analysis of 2-(2-phenylethyl)chromones by UPLC-ESI-QTOF-MS and multivariate statistical methods in wild and cultivated agarwood. Int. J. Mol. Sci..

[3] Wang S., Yu Z.X., Wang C.H., Wu C.M., Guo P., Wei J.H. (2018). Chemical constituents and pharmacological activity of agarwood and *Aquilaria* plants. Molecules.

[4] Liao G., Mei W.L., Dong W.H., Li W., Wang P., Kong F.D., Gai C.J., Song X.Q., Dai H.F. (2016). 2-(2-Phenylethyl)chromone derivatives in artificial agarwood from *Aquilaria sinensis*. Fitoterapia.

[5] National Pharmacopoeia Commission (2010). Chinese Pharmacopoeia.

[6] Li W., Cai C.H., Dong W.H., Guo Z.K., Wang H., Mei W.L. (2014). 2-(2-Phenylethyl)chromone derivatives from Chinese agarwood induced by artificial holing. Fitoterapia.

[7] Yu M., Liu Y.Y., Feng J., Chen D.L., Yang Y., Liu P.W., Yu Z.X., Wei J.H. (2021). Remarkable phytochemical characteristics of Chi-Nan agarwood induced from new-found Chi-Nan germplasm of *Aquilaria sinensis* compared with ordinary agarwood. Int. J. Anal. Chem..

[8] Wu Z.Q., Liu W.Z., Li J., Yu L.W., Lin L. (2020). Dynamic analysis of gene expression and determination of chemicals in agarwood in *Aquilaria sinensis*. J. For. Res..

[9] Xia B., Li J.R., Yang D.L., Mei W.L., Ding L.S., Zhou Y. (2014). A rapid and highly specific method to evaluate the presence of 2-(2-phenylethyl) chromones in agarwood by supercritical fluid chromatography-mass spectrometry. Eur. J. Mass Spectrom..

[10] Wang X.H., Gao B.W., Liu X., Dong X.J., Zhang Z.X., Fan H.Y., Zhang L., Wang J., Shi S.P., Tu P.F. (2016). Salinity stress induces the production of 2-(2-phenylethyl)chromones and regulates novel classes of responsive genes involved in signal transduction in *Aquilaria sinensis* calli. BMC Plant Biol..

[11] Xia L.L., Li W., Wang H., Chen H.Q., Cai C.H., Yang L., Jiang B., Yang Y.L., Mei W.L., Dai H.F. (2019). LC-MS guided identification of dimeric 2-(2-phenylethyl)chromones and sesquiterpene-2-(2-phenylethyl)chromone conjugates from agarwood of *Aquilaria crassna* and their cytotoxicity. Fitoterapia.

[12] Patil V.M., Masand N., Verma S., Masand V. (2021). Chromones: Privileged scaffold in anticancer drug discovery. Chem. Biol. Drug Des..

[13] Li C.G., Pan L., Han Z.Z., Xie Y.Q., Hu H.J., Liu X.L., Wu L.H., Yang L., Wang Z.T. (2020). Antioxidative 2-(2-phenylethyl)chromones in Chinese eaglewood from *Aquilaria sinensis*. J. Asian Nat. Prod. Res..

[14] He Q., Hu D.B., Zhang L., Xia M.Y., Yan H., Li X.N., Luo J.F., Wang Y.S., Yang J.H., Wang Y.H. (2021). Neuroprotective compounds from the resinous heartwood of *Aquilaria sinensis*. Phytochemistry.

[15] Yang L., Qiao L.R., Xie D., Yuan Y.H., Chen N.H., Dai J.G., Guo S.X. (2012). 2-(2-Phenylethyl)chromones from Chinese eaglewood. Phytochemistry.

[16] Liao G., Dong W.H., Yang J.L., Li W., Wang J., Mei W.L., Dai H.F. (2018). Monitoring the chemical profile in agarwood formation within one year and speculating on the biosynthesis of 2-(2-phenylethyl)chromones. Molecules.

[17] Li W., Chen H.Q., Wang H., Mei W.L., Dai H.F. (2021). Natural products in agarwood and *Aquilaria* plants: Chemistry, biological activities and biosynthesis. Nat. Prod. Rep..

[18] Xiao M.J., Gao Z.H., Wei J.H. (2019). Advances in research on bioactivity and biosynthesis of 2-(2-phenylethyl) chromones. Chin. Pharm. J..

[19] Ahn S., Ma C.T., Choi J.M., An S., Lee M., Le T.H.V., Pyo J.J., Lee J., Choi M.S., Kwon S.W. (2019). Adiponectin-secretion-promoting phenylethylchromones from the agarwood of *Aquilaria malaccensis*. J. Nat. Prod..

[20] Wang X.H., Gao B.W., Nakashima Y., Mori T., Zhang Z.X., Kodama T., Lee Y.E., Zhang Z.K., Wong C.P., Liu Q.Q. (2022). Identification of a diarylpentanoid-producing polyketide synthase revealing an unusual biosynthetic pathway of 2-(2-phenylethyl) chromones in agarwood. Nat. Commun..

[21] Xiao M.J., Wang B.B., Feng Y.N., Sun P.W., Rong M., Liu Y.Y., Chen D.L., Lv F.F., Gao Z.H., Wei J.H. (2022). Three candidate 2-(2-phenylethyl)chromone- producing type III polyketide synthases from *Aquilaria sinensis* (Lour.) Gilg have multifunctions synthesizing benzalacetones, quinolones and pyrones. Ind. Crops Prod..

[22] Wang X.H., Zhang Z.X., Dong X.J., Feng Y.Y., Liu X., Gao B.W., Wang J.L., Zhang L., Wang J., Shi S.P. (2017). Identification and functional characterization of three type III polyketide synthases from *Aquilaria sinensis* calli. Biochem. Biophys. Res. Commun..

[23] Das A., Begum K., Akhtar S., Ahmed R., Tamuli P., Kulkarni R., Banu S. (2023). Genome-wide investigation of Cytochrome P450 superfamily of *Aquilaria agallocha*: Association with terpenoids and phenylpropanoids biosynthesis. bioRxiv-Plant Biol..

[24] Okudera Y., Ito M. (2009). Production of agarwood fragrant constituents in *Aquilaria* calli and cell suspension cultures. Plant Biotechnol..

[25] Yang J.L., Dong W.H., Kong F.D., Liao G., Wang J., Li W., Mei W.L., Dai H.F. (2016). Characterization and analysis of 2-(2-phenylethyl)-chromone derivatives from agarwood (*Aquilaria crassna*) by artificial holing for different times. Molecules.

[26] Khadem S., Marles R.J. (2012). Chromone and flavonoid alkaloids: Occurrence and bioactivity. Molecules.

[27] Chen X.D., Zhu X.L., Feng M.R., Zhong Z.J., Zhou X., Chen X.Y., Ye W., Zhang W.M., Gao X.X. (2017). Relationship between expression of chalcone synthase genes and chromones in artificial agarwood induced by formic acid stimulation combined with *Fusarium* sp. A2 inoculation. Molecules.

[28] Mi X.Y., Feng Y.Y., Guan F.Y., Zheng Y.Y., Qiu H.L., Gao B.W., Wang B.W., Liu X., Wang J., Tu P.F. (2024). Overexpression of plant polyketide synthase *AsPECPS* from *Aquilaria sinensis* enhances the tolerance of the transgenic *Nicotiana benthamiana* to salt stress and ABA treatment. Plant Cell Tissue Organ Cult..

[29] Ding X.P., Wang H., Huang S.Z., Zhang H., Chen H.Q., Chen P.W., Wang Y.G., Yang Z., Wang Y.L., Peng S.Q. (2024). Molecular evolution and characterization of type III polyketide synthase gene family in *Aquilaria sinensis*. Plant Physiol. Biochem..

[30] Yang Y., Zhu J.H., Zeng J., Mei W.L., Dai H.F., Peng S.Q. (2023). Cloning and expression analysis of *AsOMT1* gene in *Aquilaria sinensis*. Plant Physiol. J..

[31] Wu W.L., Yan T.T., Sun X.C., Wilson I., Li G.Y., Hong Z., Shao F.J., Qiu D.Y. (2024). Identification and characterization of two *O*-methyltransferases involved in biosynthesis of methylated 2-(2-phenethyl)chromones in agarwood. J. Exp. Bot..

[32] Wang B.B., Hai Y., Zhang L., Zhang M.L., Ding N., Fan J.P., Zhang B.B., Zhang Z.K., Wang J., Wang X.H. (2024). Identification of *O*-methyltransferases potentially contributing to the structural diversity of 2-(2-phenylethyl)chromones in agarwood. J. Agric. Food Chem..

[33] Wang Y.X., Huang W.Q., Tian W.S., Mo T., Yan Y.R., Cui X.X., Liu X. (2023). Enzymatic biosynthesis of novel 2-(2-phenylethyl)chromone glycosides catalyzed by UDP-glycosyltransferase UGT71BD1. Biochem. Biophys. Res. Commun..

[34] Dong X.J., Gao B.W., Feng Y.Y., Liu X., Wang J., Wang J.L., Tu P.F., Wang X.H., Shi S.P. (2018). Production of 2-(2-phenylethyl)chromones in *Aquilaria sinensis* calli under different treatments. Plant Cell, Tissue Organ Cult..

[35] Zhao T., Zhou Y., Li W.Y., Ma X.Y., Zhan R.T., Chen W.W. (2019). Experimental induction of 2-(2-phenylethyl) chromone in aerial roots of *Aquilaria sinensis*. Pak. J. Bot..

[36] Tan C.S., Isa N.M., Ismail I., Zainal Z. (2019). Agarwood induction: Current developments and future perspectives. Front. Plant Sci..

[37] Ma S., Fu Y.L., Li Y.J., Wei P.L., Liu Z.G. (2021). The formation and quality evaluation of agarwood induced by the fungi in *Aquilaria sinensis*. Ind. Crops Prod..

[38] Grotewold E. (2005). Plant metabolic diversity: A regulatory perspective. Trends Plant Sci..

[39] Zhang H., Ding X.P., Wang H., Chen H.Q., Dong W.H., Zhu J.H., Wang J., Peng S.Q., Dai H.F., Mei W.L. (2023). Systematic evolution of *bZIP* transcription factors in Malvales and functional exploration of *AsbZIP14* and *AsbZIP41* in *Aquilaria sinensis*. Front. Plant Sci..

[40] Dröge-Laser W., Weiste C. (2018). The C/S1 bZIP network: A regulatory hub orchestrating plant energy homeostasis. Trends Plant Sci..

[41] Yang Y., Zhu J.H., Wang H., Guo D., Wang Y., Mei W.L., Peng S.Q., Dai H.F. (2023). Systematic investigation of the R2R3-MYB gene family in *Aquilaria sinensis* reveals a transcriptional repressor AsMYB054 involved in 2-(2-phenylethyl)chromone biosynthesis. Int. J. Biol. Macromol..

[42] Vranová E., Coman D., Gruissem W. (2012). Structure and dynamics of the isoprenoid pathway network. Mol. Plant..

[43] Yang Z., Mei W.L., Wang H., Zeng J., Dai H.F., Ding X.P. (2023). Comprehensive analysis of *NAC* Transcription Factors Reveals Their Evolution in Malvales and Functional Characterization of *AsNAC019* and *AsNAC098* in *Aquilaria sinensis*. Int. J. Mol. Sci..

[44] Mei W.L., Zuo W.J., Yang D.L., Dong W., Dai H.F. (2013). Advances in the mechanism, artificial agarwood-induction techniques and chemical constituents of artificial agarwood production. Chin. J. Trop. Crops.

[45] Yan T.T., Yang S., Chen Y., Wang Q., Li G.Y. (2019). Chemical profiles of cultivated agarwood induced by different techniques. Molecules.

[46] Wu W.L., Sun X.C., Wilson I., Jiang L.Y., Jiang X.Y., Shao F.J., Qiu D.Y. (2024). Effects of ferrous sulfate and methyl jasmonate treatment on the content of 2-(2-phenethyl)chromones in *Aquilaria sinensis*. Plant Cell Tissue Organ Cult..

[47] Nguyen H.T., Min J.E., Long N.P., Thanh M.C., Le T.H.V., Lee J., Park J.H., Kwon S.W. (2017). Multi-platform metabolomics and a genetic approach support the authentication of agarwood produced by *Aquilaria crassna* and *Aquilaria malaccensis*. J. Pharm. Biomed. Anal..

[48] Ding X.P., Mei W.L., Lin Q., Wang H., Wang J., Peng S.Q., Li H.L., Zhu J.H., Li W., Wang P. (2020). Genome sequence of the agarwood tree *Aquilaria sinensis* (Lour.) Spreng: The first chromosome-level draft genome in the Thymelaeceae family. GigaScience.

[49] Nong W.Y., Law S.T.S., Wong A.Y.P., Baril T., Swale T., Chu L.M., Hayward A., Lau D.T.W., Hui J.H.L. (2020). Chromosomal-level reference genome of the incense tree *Aquilaria sinensis*. Mol. Ecol. Resour..

[50] Xu Y.H., Zhang Z., Wang M.X., Wei J.H., Chen H.J., Gao Z.H., Sui C., Luo H.M., Zhang X.L., Yang Y. (2013). Identification of genes related to agarwood formation: Transcriptome analysis of healthy and wounded tissues of *Aquilaria sinensis*. BMC Genom..

[51] Ye W., Wu H.Q., He X., Wang L., Zhang W.M., Li H.H., Fan Y.F., Tan G.H., Liu T.M., Gao X.X. (2016). Transcriptome sequencing of chemically induced *Aquilaria sinensis* to identify genes related to agarwood formation. PLoS ONE.

[52] Wang X.H., Dong X.J., Feng Y.Y., Liu X., Wang J.L., Zhang Z.X., Li J., Zhao Y.F., Shi S.P., Tu P.F. (2018). H_2_O_2_ and NADPH oxidases involve in regulation of 2-(2-phenylethyl) chromones accumulation during salt stress in *Aquilaria sinensis* calli. Plant Sci..

[53] Ye W., Zhang W.M., Liu T.M., Zhu M.Z., Li S.N., Li H.H., Huang Z.L., Gao X.X. (2018). iTRAQ-based auantitative proteomic analysis of chemically induced *Aquilaria sinensis* provides insights into agarwood formation mechanism. Proteomics.

